# A Non-Invasive Multichannel Hybrid Fiber-Optic Sensor System for Vital Sign Monitoring

**DOI:** 10.3390/s17010111

**Published:** 2017-01-08

**Authors:** Marcel Fajkus, Jan Nedoma, Radek Martinek, Vladimir Vasinek, Homer Nazeran, Petr Siska

**Affiliations:** 1 Department of Telecommunications, Faculty of Electrical Engineering and Computer Science, VSB–Technical University of Ostrava, 17 Listopadu 15, Ostrava 70833, Czech Republic; jan.nedoma@vsb.cz (J.N.); vladimir.vasinek@vsb.cz (V.V.); petr.siska@vsb.cz (P.S.); 2 Department of Cybernetics and Biomedical Engineering, Faculty of Electrical Engineering and Computer Science, VSB-Technical University of Ostrava, 17 Listopadu 15, Ostrava 70833, Czech Republic; radek.martinek@vsb.cz; 3 Department of Electrical and Computer Engineering, University of Texas El Paso, 500 W University Ave, El Paso, TX 79968, USA; hnazeran@utep.edu

**Keywords:** vital signs, heart rate (HR), respiratory rate (RR), body temperature, fiber Bragg grating (FBG), polydimethylsiloxane (PDMS), magnetic resonance imaging (MRI), non-invasive measurements, patient monitoring, biomedical engineering

## Abstract

In this article, we briefly describe the design, construction, and functional verification of a hybrid multichannel fiber-optic sensor system for basic vital sign monitoring. This sensor uses a novel non-invasive measurement probe based on the fiber Bragg grating (FBG). The probe is composed of two FBGs encapsulated inside a polydimethylsiloxane polymer (PDMS). The PDMS is non-reactive to human skin and resistant to electromagnetic waves, UV absorption, and radiation. We emphasize the construction of the probe to be specifically used for basic vital sign monitoring such as body temperature, respiratory rate and heart rate. The proposed sensor system can continuously process incoming signals from up to 128 individuals. We first present the overall design of this novel multichannel sensor and then elaborate on how it has the potential to simplify vital sign monitoring and consequently improve the comfort level of patients in long-term health care facilities, hospitals and clinics. The reference ECG signal was acquired with the use of standard gel electrodes fixed to the monitored person’s chest using a real-time monitoring system for ECG signals with virtual instrumentation. The outcomes of these experiments have unambiguously proved the functionality of the sensor system and will be used to inform our future research in this fast developing and emerging field.

## 1. Introduction

Current trends in the development of vital sign monitoring technologies demonstrate that the future points in the direction of the introduction of sophisticated diagnostic devices that integrate several diagnostic measures in one all-purpose instrument or probe.

Here, we introduce the concept of a novel sensor system, which offers the potential of continuously monitoring the vital signs of up to 128 individuals by using a fiber optic probe. This patent-pending probe, which functions on the basis of the fiber Bragg grating, was designed, constructed, and validated by the authors in the Czech Republic. The advantage of the proposed multichannel fiber-optic sensor system is the possibility of monitoring patients in the harsh magnetic resonance (MR) environment (e.g., Magnetic Resonance, X-ray, UV absorption and radiation, etc.).

The authors have developed a measurement probe that allows for monitoring the mechanical vibrations of human body evoked by living activities: breathing and heart rate [1]. The body temperature as well as the respiratory and heart rate were obtained by a spectral evaluation of the measured signals. The mechanical strain is transferred to the fiber Bragg grating (FBG) by a thoracic elastic strap. This monitoring method is fully isolated, and it ensures absolute electrical safety for the patient.

Recent years have witnessed a growing increase in the utilization of Optical Sensors (OS) for a variety of emerging biomedical applications [2,3]. A number of articles [4,5,6,7,8,9,10,11,12,13,14,15,16,17,18,19,20,21,22,23,24,25,26,27,28,29,30,31,32] presented only results for measurement of respiration rate or heart rate. For example, the article [7] reports on results obtained from monitoring the respiration and cardiac activity of a patient during a magnetic resonance imaging (MRI) survey using an optical strain sensor based on an FBG. Several research groups reported vital signs monitoring based on fiber interferometry method [14,26,27,28,29]. FBG sensors were used to monitor the cardiac activity and respiration [4,6,8,10,13,17,19,28,30].

Textiles can be used to encapsulate sensors and measure heartbeat and respiration rate simultaneously. Several techniques, such as wrap and weft knitting, weaving and stitching, can be used to embed the fiber sensing elements into textile fabrics [8,9,16,21,31,32]. For example, the textile based respiratory rate sensor demonstrated in [21] is very simple, cost-effective and comfortable to wear. This sensor can be placed on thoracic or abdominal areas, but the heartbeat signal cannot be measured due to the lower sensitivity of fiber macro bend effects. However, the authors in [9] report on a textile fiber optic microbend sensor for heartbeat and respiration monitoring simultaneously.

Our novel measurement probe (described below) allows measurement of body temperature in addition to other advantages offered by the sensors described in the above articles. Based on our comprehensive literature and patent search, we can safely claim that our presented solution is innovative.

In general, there are three types of Optical Sensors: (1) non-invasive sensors, which come into direct contact with the human skin; (2) minimally-invasive sensors, which are used for measurements carried out in body cavities; and (3) invasive sensors, which are used for measurements made inside organs or in the bloodstream (intravascular). Desirable characteristic features of Optical Sensors include their independence from an active power supply and a high immunity to electromagnetic interference. Thanks to these attributes, Optical Sensors can be used with other electronic equipment without generating electric noise that may compromise the quality of vital sign monitoring and potentially lead into patient safety concerns. Optical Sensors are gaining more popularity due to their flexibility, improved functionality, and reliability. The very small dimensions of optical fibers allow them to be encapsulated inside very thin catheters and injection needles, thereby enabling localized and minimally-invasive monitoring. Biocompatibility is a very important consideration in the acquisition and evaluation of high quality data from sensors coming into contact with living tissue. As Optical Sensors are biocompatible and do not influence the patient’s body in any major way, they offer a great level of patient comfort during vital sign monitoring.

The major aim of this article is to introduce our novel vital sign measurement probe (which belongs to the group of non-invasive optical sensors) and its associated sensor system. In this limited space, we do not intend to compare our system with other existing sensors. The comprehensive characterization of our novel sensor system and its comparison with other existing sensors will be the focus of our future articles with detailed signal processing.

## 2. Methods

### 2.1. Fiber Bragg Grating

**Figure 1:**
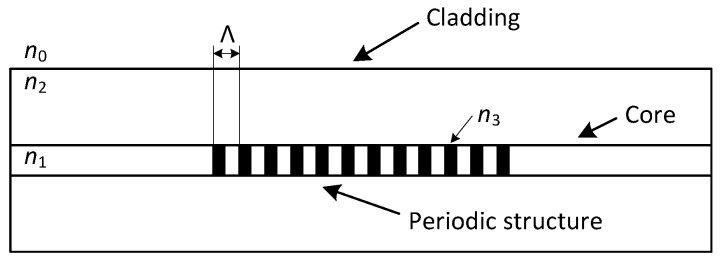
The structure of fiber Bragg grating.

The FBG is characterized by a periodic change of the refractive index in the fiber’s core (Figure 1). When we project a beam of light from a Super-luminescent Light Emitting Diode (SLED) into an optical fiber, a narrow spectral part of the beam is reflected while other wavelengths are transmitted without any attenuation by this structure. The reflected wavelength is called the Bragg wavelength ($\lambda_{B}$) and is given by the following equation:

(1) $$\lambda_{B}=2n_{eff}\Lambda ,$$

where $n_{eff}$ is the effective refractive index of the fiber’s core within the Bragg grating and Λ is the period of its changes.

External effects like deformation or temperature influence the Bragg wavelength and the period of changes of the refractive index in a linear fashion, which translates into a linear shift in the Bragg wavelength. To describe the Bragg grating sensitivity in a quartz optical fiber, we use the normalized deformation coefficient (at constant temperature) as:

(2) $$\frac{1}{\lambda_{B}}\frac{\Delta \lambda_{B}}{\Delta \varepsilon }=0.78\times 10^{-6}\;\mu {\text{strain}}^{-1},$$

and the normalized temperature coefficient (at constant strain) as:

(3) $$\frac{1}{\lambda_{B}}\frac{\Delta \lambda_{B}}{\Delta T}=6.678\times 10^{-6}\;{}^{\circ }C^{-1},$$

where $\lambda_{B}$ is the Bragg wavelength, $\Delta \lambda_{B}$ is the shift of the Bragg wavelength, Δε is the change of deformation and ΔT represents a change in temperature. The FBG with the Bragg wavelength at 1500 nm shows the deformation sensitivity of 1.2 pm/μstrain and temperature sensitivity of 10.3 pm/°C [33].

**Figure 2:**
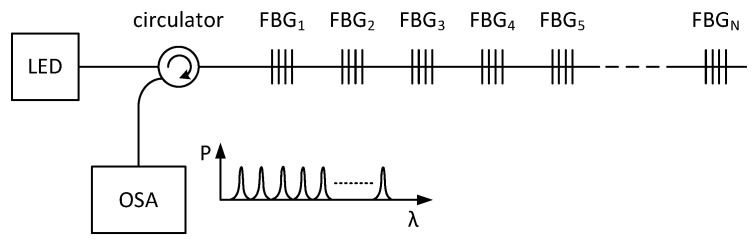
A schematic diagram of the Wavelength Division Multiplexing (WDM) with Light Emitting Diode (LED), Fiber Bragg Gratings (FBGs) and Optical Spectrum Analyzer (OSA).

As FBGs are single-point sensors, with multiplexing techniques, we can easily connect them together and obtain a multi-point measurement probe. The most common methods are the wavelength-division multiplexing (WDM) and time-division multiplexing (TDM). The analysis of the capacity of these multiplexing methods is described in [34]. The most widely used multiplexing method in sensor applications is the wavelength-division multiplexing, which is based on the spectral division of individual gratings. In the WDM method, light from an LED passes through a circulator to the FBG array, and the reflected light is detected in the OSA (Optical Spectrum Analyser), where each FBG is tuned to a different Bragg wavelength (Figure 2). In the OSA, each peak represents one FBG, and the respective frequency shifts are related to the applied deformation or temperature change. The measured value is expressed by a shift of the Bragg wavelength of the FBG that is being affected.

To apply the wavelength-division multiplexing method, it is necessary to avoid the overlapping of neighboring spectrums. For this reason, each FBG probe is assigned a specific measurement window, whose size is given by a sensitivity coefficient as well as the expected maximal influence of the measured value.

### 2.2. Novel Design of Measurement Probe

**Figure 3:**
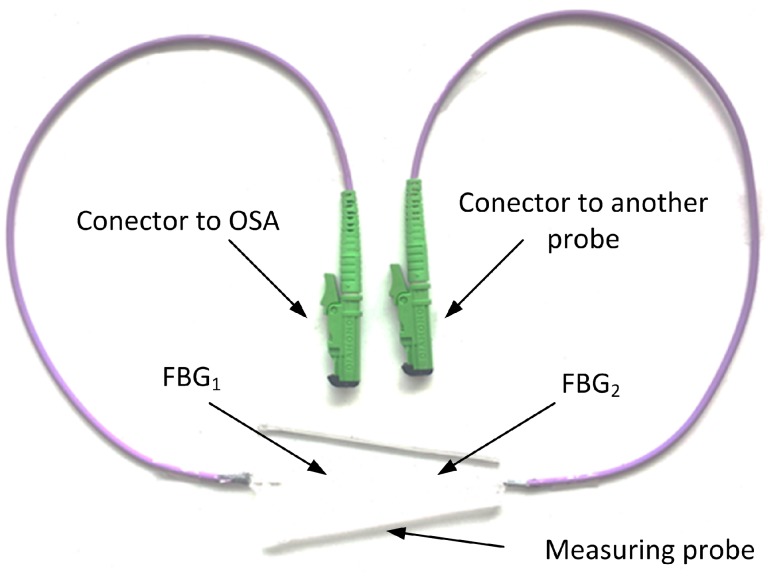
The designed and realized probe.

Our novel measurement probe is based on two FBGs encapsulated inside a polydimethylsiloxane (PDMS) polymer (Figure 3). As FBGs are sensitive to strain and temperature changes, they are suitable for many biomedical measurements. For example, they could be used in thermodilution-based cardiac output monitoring instruments or temperature measurement systems.

**Figure 4:**
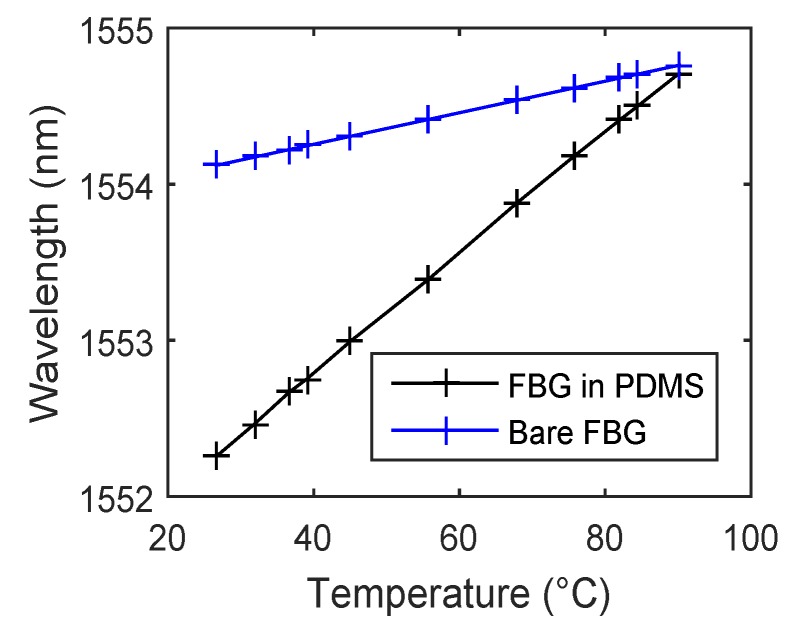
The temperature sensitivity of a bare Fiber Bragg Grating (FBG) and its encapsulated version inside the polydimethylsiloxane (PDMS) polymer.

We chose the PDMS polymer to increase the temperature sensitivity of our probe and ensure its non-reactivity to the human skin. Figure 4 shows temperature sensitivity measurements of a bare FBG at a Bragg wavelength of 1554.1203 nm and its encapsulated version inside the PDMS as an example. It is evident that the temperature sensitivity significantly increases (almost four times) from 10.378 pm/°C to 39.44 pm/°C due to encapsulation in the PDMS polymer. The presented results in [35] indicate that this type of encapsulation does not affect the structure of the FBG.

The encapsulation of the measurement probe was realized by PDMS with the designation of Sylgard 184. Sylgard 184 is a two-component casting compound: the A component creates its own pre-polymer and the B component is a curing agent. Both components are mixed together according to a datasheet in a weight ratio of 10:1 (A:B). Bubbles and microbubbles that result from the combination of the pre-polymer and the curing agent can be removed using an ultrasonic bath. Homogeneity of the connection is realized using a laboratory shaker. The measurement probe contains two connectors of EURO 2000 type. The first connector is plugged into the measurement system or a preceding probe, and the second one is plugged into a succeeding probe.

### 2.3. The Multichannel Concept

**Figure 5:**
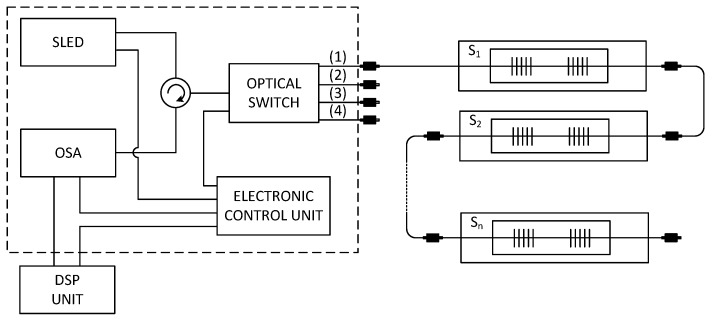
The measurement scheme of our multichannel hybrid fiber-optic sensor system.

The multichannel hybrid fiber-optic sensor system (Figure 5) is based upon a wide-spectral SLED as the optical source with a bandwidth from 1512.5 nm to 1587.5 nm and an output power of 1 mW. The emitting light is projected by means of a circulator into a four-channel optical switch with the switching time about 0.5 ms. This switch routes incoming light rays (from the SLED) into four measurement channels. This arrangement increases the sensor capacity up to four times. One measurement channel can store up to 32 vital sign measurement probes, where each probe is assigned a measurement window with a spectral width around 1.9 nm (Table 1).

Reflected signals from each measurement channel are projected through the circulator into an optical spectrum analyzer. The signal is then processed in the Digital Signal Processing (DSP) Unit. The Electronic Control Unit controls the parameters of each individual optical unit such as the capacity of the light source, the switching speed of the optical switch and the sensitivity of the optical spectrum analyzer.

The experimental solution proposed in this article uses the fiber Bragg gratings encapsulated into PDMS polymer to monitor patient’s vital signs. Individual sensors are detected by one optical fiber. To distinguish individual readings from FBGs the wavelength division multiplexing method is used. Each FBG sensor is assigned a specific measurement window. The fiber Bragg gratings of individual sensors belonging to a specific type of spectral model [36] are given by the relationship:

(4) $$\lambda_{Bn}=\frac{\lambda_{B0}{\left(1+k_{N\varepsilon }MR_{P}\right)}^{n-1}}{{\left(1-k_{N\varepsilon }MR_{N}\right)}^{n}}+GB\sum_{i=0}^{n-2}\frac{{\left(1+k_{N\varepsilon }MR_{P}\right)}^{n-2-i}}{{\left(1-k_{N\varepsilon }MR_{N}\right)}^{n-1-i}},$$

where $\lambda_{Bn}$ is a Bragg wavelength of the *n*-th sensor, $\lambda_{B0}$ is a wavelength of the left boundary of spectral region’s radiation source, $k_{N\varepsilon }$ is a normalized deformation coefficient, $MR_{P}$ is a positive measurement scope, $MR_{N}$ is a negative measurement scope and GB is a Guard Band. The maximum number of sensors that can be evaluated from one optic fiber is limited by the right boundary of the radiation source (the right boundary of the last measurement window must be smaller than the right boundary of the radiation source).

Movements of the thoracic cavity (its expansion or contraction) depend on the way of breathing (deep or shallow) and particularly on its capacity. Due to this fact, a series of measurements were carried out to set the input parameters ($MR_{P}$, $MR_{N}$, GB) of the sensory array design. The aim of this measurement was to specify an extreme case which involves the largest expansion of the thoracic cavity during breathing, which can cause the deformation of the sensor. These measurements were carried out in 10 volunteer test subjects of different age, sex, weight and height. Based on the realized measurements, the input parameters were defined: $MR_{P}=800$ μstrain and $MR_{N}=-800$ μstrain. The Guard Band provides a level of immunity against unpredictable effects. However, it decreases with the number of monitored subjects. In our computations we selected a GB = 0.4 nm based on a number of experimental observations.

Table 1 shows the parameters of the fiber Bragg gratings for each sensor. These parameters were calculated by using Equation (4) with input parameters $MR_{P}=800$ μstrain, $MR_{N}=-800$ μstrain, GB=0.4 nm, $\lambda_{B0}=1512.5$ nm, $k_{N\varepsilon }=0.78\times 10^{-6}\;\mu {\text{strain}}^{-1}$.

**Figure 6:**
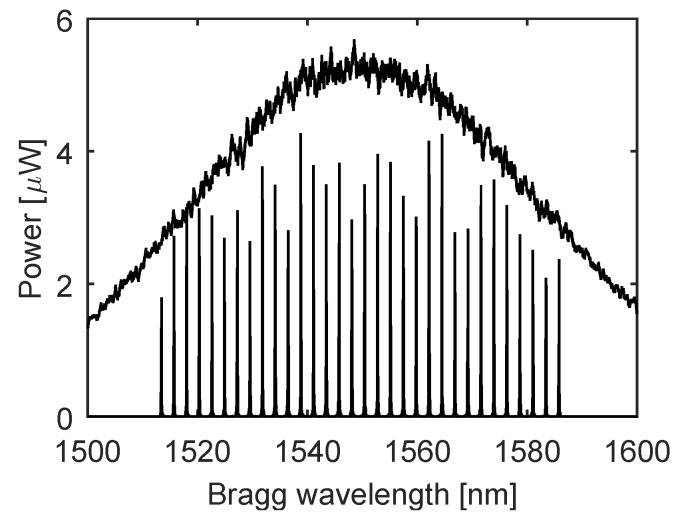
The spectrum of the Light Emitting Diode (LED) output radiation (upper graph) and the spectral distribution of the fiber Bragg gratings in the sensor array (lower graph).

Figure 6 shows the power spectrum of LED source radiation with the reflected spectral parts of 32 FBG sensors in series.

## 3. Results

**Figure 7:**
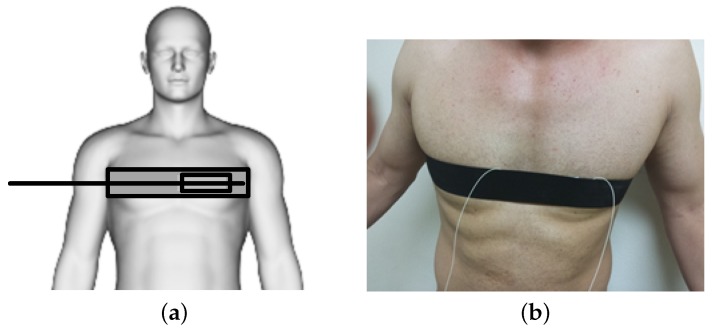
(**a**) A schematic diagram of the experimental set up. (**b**) Experimental set up to acquire vital sign data from a human subject using the novel probe embedded in a thoracic elastic strap.

The experimental tests were carried out on ten persons of both sexes (six men and four women) in a room with the temperature of 24 °C. The tested persons were between 20 and 45 years of age, their weight was between 55 and 115 kg and their height was between 155 and 192 cm. No significant differences were found in the quality of the received signal depending on the age, weight, and height. The experiments were carried out in a lab environment and they were discussed with the senior doctor of the long-term health care department of the University Hospital in Brno, Czech Republic. The vital sign monitoring probe was placed around the pulmonic area on the chest and fixed into the position by a contact elastic strap (Figure 7). The subjects were tested in both standing and supine positions. The measurements showed that the testing positions did not influence the sensitivity of the measurement probe.

Two approaches were used to evaluate the heart and respiratory rates. The first approach allows for the determination of these rates in the time domain by identifying the periodic cycles in the form of local maximum Equation (5). Whereas in the second approach, the Fourier transform is applied on obtained measurements to calculate the dominant frequency *f* and produce the rates in cycles or respirations per minute (rpm) by using Equation (6).

(5) $$rate=\frac{60}{t_{i}-t_{i-1}},$$

(6) rate=60f,

where $t_{i}$ is a time position of the *i*-th maximum (of breathing and pulse progress), $t_{i-1}$ is a time position of the previous maximum, and *f* is a dominant frequency obtained using the Fourier transform.

The measurements are based on monitoring the movements of the thoracic cavity of a subject while breathing. The mechanical strain is transferred to the FBG by a thoracic elastic strap. The body temperature as well as respiratory and heart rates were obtained by the spectral evaluation of the measured signals.

### 3.1. Measurement of Respiratory Rate

An application software was developed in Matlab (R2015a, MathWorks, Natick, MA, USA) to perform a variety of tasks including: (1) calculation of the breathing rate based upon the acquired breathing data, (2) controlling the measurement process and setting up the constant parameters necessary to for breathing rate calculations, (3) carrying out the final analysis and evaluation of the breathing rate measurement error. The software also produced a bar chart which was updated based upon previously set parameters such as duration of the measurement and durations of breathing in (inspiration) and breathing out (expiration). The bar charts clearly validated that the subject under test was breathing and ensured the determination of a relatively constant breathing rate. The application also produced the final value of the respiratory rate, which was used for data processing and determination of the system error rate.

**Figure 8:**
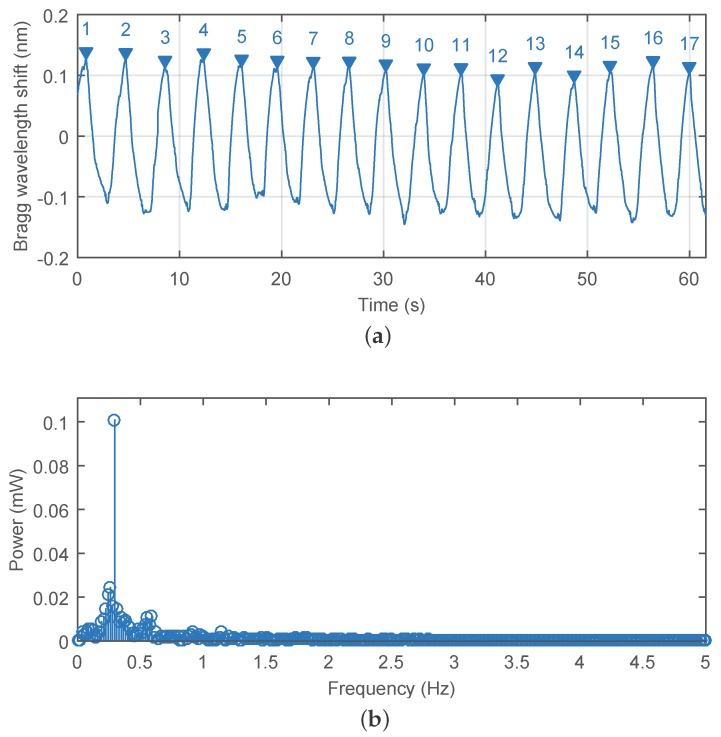
The Bragg wavelength shift (**a**) and frequency spectrum (**b**) during monitoring of the subject’s M1’s breathing activity.

The multichannel measurement system (including our novel probe) was used to monitor the subject’s breathing activity (measured as cyles or respirations per minute) with a sampling frequency of 300 Hz. For example, the Bragg wavelength shift for the subject M1 is shown in Figure 8a. Using Fourier series analysis (as we are analyzing a periodic continuous waveform), it was possible to determine a dominant frequency of the the first harmonic (0.295 Hz) of the breathing activity and to calculate a respiratory rate of 17.7 cycles/min (Figure 8b). The respiratory rate (RR) is expressed in respirations per minute (rpm).

**Figure 9:**
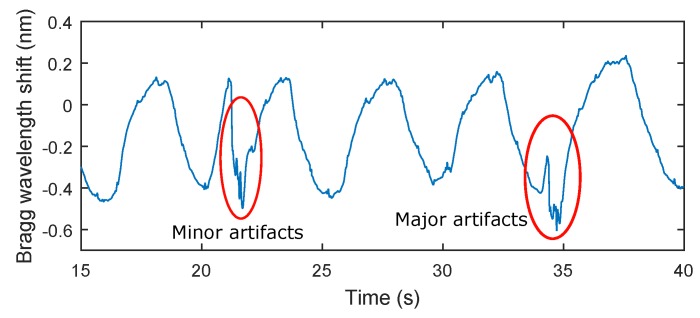
A recording from a subject in this study (M2). Subject M2 was asked to change his position and to simulate strong coughing. For example, the influence of a position change on the respiratory rate is shown in **red** on the left (minor artifacts) and the influence of strong coughing on the breathing rate is shown in **red** on the right (major artifacts).

The key experimental results of breathing measurements are summarized in Table 2. The maximum relative error of 5.41% was observed in patient F3. The total relative system error rate for ten test subjects and total time record of 159 min and 40 s was 3.9%. It should be noted that in the monitoring of mechanical vibrations of human body, as in any physiological measurement, the signal quality can be corrupted by external motion artifacts. These errors can be caused by the high sensitivity of the sensor. This sensitivity becomes problematic when the monitored person for example changes his/her position (minor artifacts), performs additional movements, coughs, etc. (major artifacts). The amplitude of stresses produced by such movements can then be higher than the amplitude of vibrations depending on breathing (Figure 9).

### 3.2. Measurement of Heart Rate

**Figure 10:**
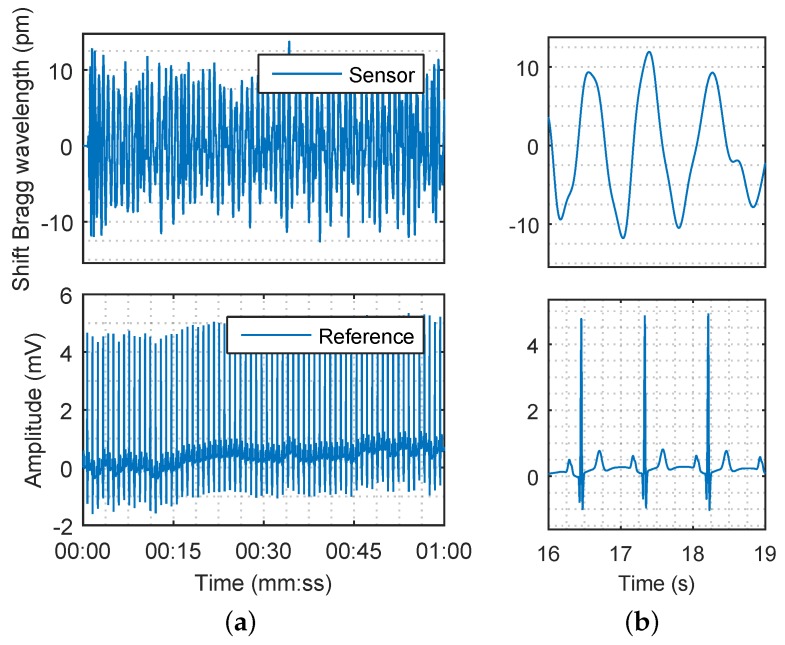
(**a**) The Bragg wavelength shift from sensor corresponding to heart rate (**top**) and Electrocardiography (ECG) reference signal originating from the cardiac electrical activity (**bottom**) for 60 s; (**b**) The detail of the signals for 3 s.

The acquired data for the blood pressure heart rate measurement were processed by using a Butterworth second order band-pass filter with corner frequencies $f_{L}$ = 0.75 Hz and $f_{H}$ = 5 Hz, respectively. The respiratory rate, which was below 0.75 Hz, and the unwanted signals (noise) with characteristic frequencies higher than 5 Hz were filtered out. The filtered signal of the subject M1 which represents the superposition of breathing and pulse pressure is shown in Figure 10a (details are shown in Figure 10b). The filtering procedure does not cause a loss of physiological information.

**Figure 11:**
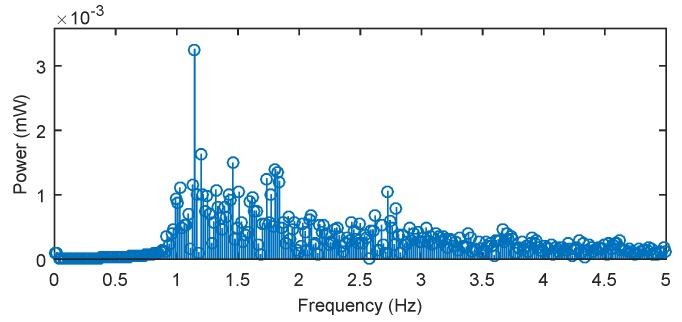
The frequency spectrum of pulsed pressure filtered signal (for heart rate calculation) in subject M1.

Using Fourier series analysis (Figure 11), we determined a dominant frequency (1.145 Hz) of the filtered (blood pressure) pulse waveform and calculated a heart rate of 68.7 beats/min, which closely matched reference measured value 68.1 beats/min. The reference ECG signal was acquired with the use of standard gel electrodes fixed to the monitored person’s chest using a real-time monitoring system for an ECG signal with virtual instrumentation. The low noise ECG signal was acquired by the National Instrument Educational Laboratory Virtual Instrumentation Suite (NI ELVIS, II Series, National Instruments, Austin, TX, USA) using a three-lead system. Appropriate signal processing was implemented on pulse pressure data acquired from our novel sensor. These processing steps produced a noise-free signal. Fast and reliable detection of pulse peaks were then accurately achieved and estimation of clinically important parameters such as pulse peak-to-peak intervals corresponding to ECG R-R intervals (R-R is the time elapsing between two consecutive R waves in the electrocardiogram) became possible.

**Figure 12:**
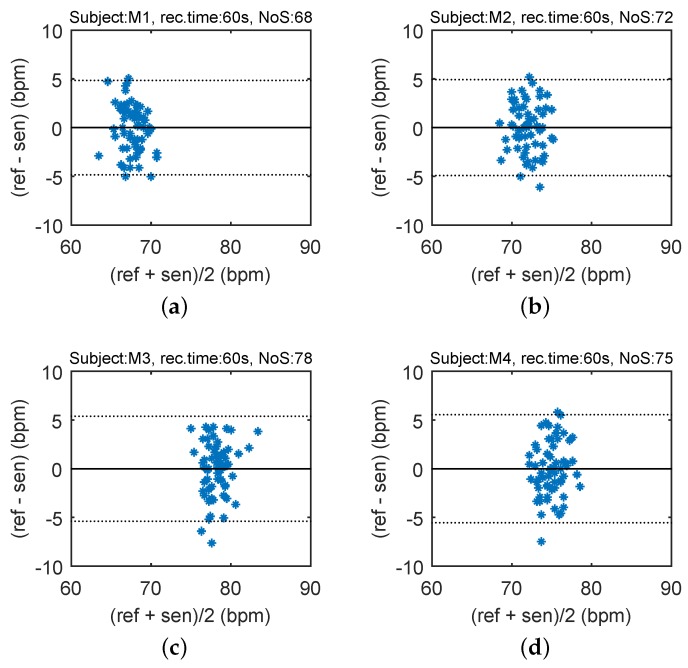

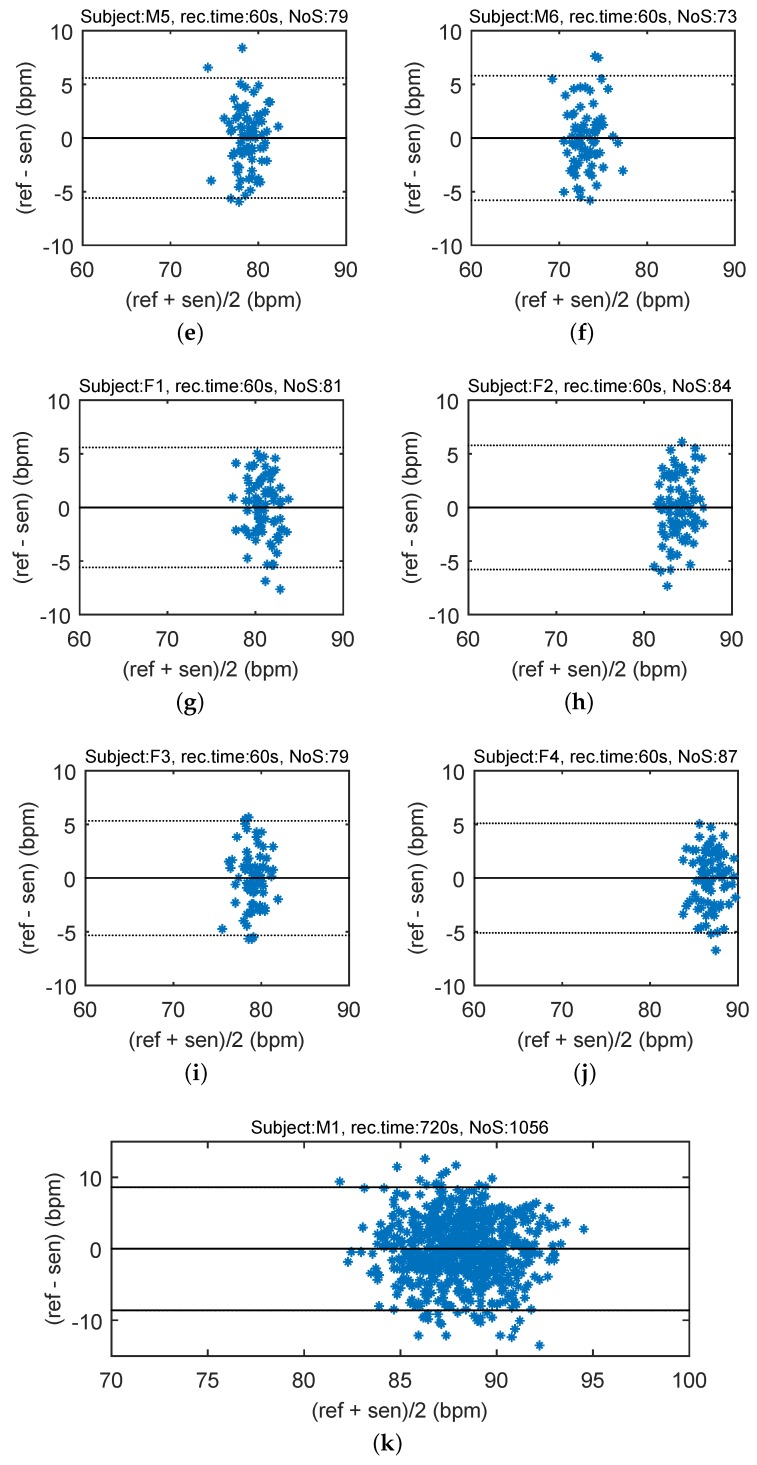
Reproducibility of HR determination capabilities of our novel sensor probe (based on comparison with ECG-based HR calculations) using the Bland-Altman method using data acquired from ten subjects.

In order to compare the differences between the reference heart rate (HR) and heart rhythm estimated using the signal from the sensor, the Bland–Altman plot was utilized [37]. The differences between the sensor and the reference traces, $x_{1}-x_{2}$, are plotted against the average, $(x_{1}+x_{2})/2$. The reproducibility is considered to be good if 95% of the results lie within a ±1.96 SD (Standard Deviation) range. Figure 12 shows the Bland–Altman statistics for four tested subjects. The heart rate (HR) is expressed in beats per minute (bpm).

**Figure 13:**
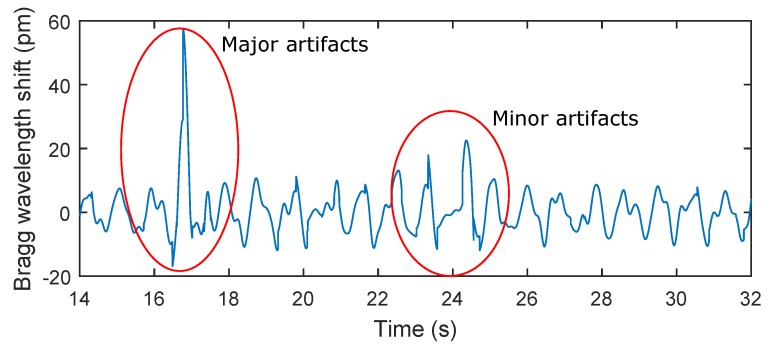
A recording from a female subject in this study. Subject F3 was asked to simulate strong coughing and to change her position. For example, the influence of strong coughing on the heart rate is shown in **red** on the left (major artifacts) and the influence of a position change on the heart rate is shown in **red** on the right (minor artifacts).

The key experimental results of cardiac activity measurements are summarized in Table 3. For the entire data set, 96.54% of the values lie within the ±1.96 SD range in HR determination, and no significant differences between individuals were observed. The results show no systematic errors, and the error has no proportional character as well as does not depend on the HR value. The Bland–Altman statistical analysis demonstrates the HR detection with a satisfactory accuracy for multiple subjects. The heart rate (HR) is expressed in beats per minute (bpm). The amplitude of stresses produced by such movements can be several dozen times higher than the amplitude produced by the heart rate (Figure 13).

### 3.3. Measurement of Body Temperature

As the FBG is sensitive to both temperature and deformation, one possible way to detect both simultaneously is to use two FBGs with different thermal and deformation sensitivitities [38]. Our novel measurement probe with its encapsulation and specific shape enables us to achieve different sensitivities. When the measurement probe is influenced by both temperature and deformation simultaneously, then the amplitude of both impacts is calculated by the following relationship:

(7) $$\left(\begin{array}{c} \Delta T\\ \Delta \varepsilon \end{array}\right)=D\left(\begin{array}{cc} K_{2\varepsilon } & -K_{1\varepsilon }\\ -K_{2T} & K_{1T} \end{array}\right)\left(\begin{array}{c} \Delta \lambda_{B1}\\ \Delta \lambda_{B2} \end{array}\right),$$

where ΔT is the temperature change, Δε is deformation, $K_{n\varepsilon }$ is the deformation coefficient, $K_{nT}$ the temperature coefficient belonging to the first or second FBG and *D* is the discriminant which must be non-zero and is given in the following equation:

(8) $$D=\frac{1}{K_{1T}K_{2\varepsilon }-K_{2T}K_{1\varepsilon }}.$$

**Figure 14:**
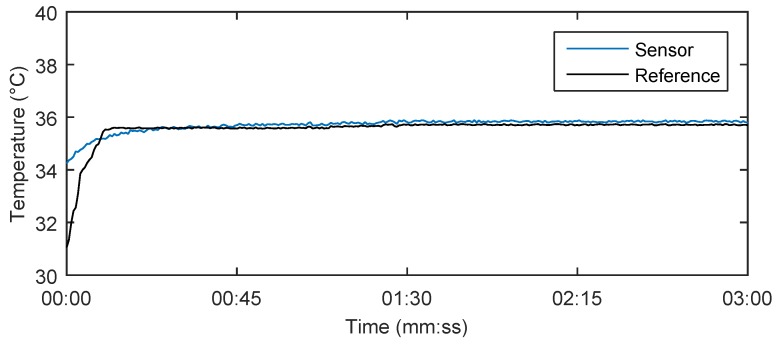
Body temperature measurement result in the test subject M1 for 180 s using our novel sensor system and reference digital thermometer.

Using the above-mentioned Equations (7) and (8), it is possible to determine the extent of deformation and temperature from the two FBG wavelengths. Figure 14 shows the results of the body temperature measurement using our system in a test subject for 180 s. The calculation of the body temperature using Equations (7) and (8) produced 35.8 °C, which closely matched the reference measured value 35.7 °C by a digital thermometer (Greisinger, Prague, Czech Republic) and its temperature recordings. The measurement accuracy is defined by a spectral distinction of the optical spectrum analyser to 1 pm. Due to a temperature sensitivity of the Fiber Bragg gratings around 10 pm/°C, the final temperature value can be determined at one-tenth of °C. When the measurements were taken, there was no record of any more significant influence (the maximum relative error was 0.36%) of body movement on the temperature measurement. This fact is based on a situation in which both gratings were under the influence of the same deformation. In this case, temperature is calculated according to Equation (7) regardless of acting deformations.

Table 4 shows the temperature values obtained in a time interval of 180 s with a step of 30 s. The maximum relative error 0.36% was observed in patient F3.

## 4. Discussion

Our novel probe and its associated multichannel system offer the possibility of continuous monitoring of the basic vital signs (Body Temperature, Heart Rate and Respiratory Rate) in up to 128 patients altogether. The creation of a totally non-invasive, electrically safe (can be used in the magnetic resonance imaging environment), cost-effective, and patient-friendly technology that enables large scale vital sign monitoring, and data collection, and analysis is indeed exciting. Such technology could prove very useful for long-term health care facilities, hospitals and clinics. Our novel non-invasive biocompatible measurement probe ensures maximum electrical safety and patient comfort. The functionality of the proposed system was verified by a series of real experimental measurements of basic vital signs.

Due to space limitation, we do not intend to consider all aspects of the design and implementation of our novel sensor system here. This short article should serve as a gateway into and an initial step towards the introduction of a new field of non-invasive and cost-effective patient monitoring which is in its infancy and has not been comprehensively explored. For any new biomedical technology like our novel sensor system and its underlying methodologies to qualify as an effective scientific instrument, and more importantly, gain clinical acceptance and find daily utilization in common clinical practice, it is essential to carry out extensive clinical research to validate its safety and efficacy. These important requirements compel us to set comprehensive research goals for clinical trials in the near future.

It is important to emphasize that the PDMS offers a unique set of desirable characteristics suitable for biomedical applications. Its non-reactivity to human skin, its mechanical and thermal resistance, its immunity to electromagnetic noise, and its ability to increase the temperature sensitivity make the PDMS a very attractive material of choice for our novel system.

However, one of the possible and current ways of non-invasive monitoring methods of vital signs of the human body is the use of patch monitors [39,40] or sensors embedded into a bed or seat that do not require additional actions to prepare the patient for monitoring [3].

The team of authors offers a solution which is basically focused on the monitoring of long-term ill patients with a minimum of physical movement load. During experiments, all test subjects were asked to simulate their natural behavior in the most accurate way (for instance, the focus was on the use of fine motor skills—not only movements of arms and hands, legs and feet, changes of body positions, coughing, but also walking). These aspects are taken into account in the results described above of the probe efficiency, or, more precisely, of the whole measurement system. Authors are ready to carry out a detailed analysis of the influence of these artifacts in the follow-up research.

## 5. Conclusions

In this article, we briefly described the overall design and realization of the prototype of a novel non-invasive multichannel hybrid fiber-optic sensor system for basic vital sign monitoring. Once clinically tested and validated, it has a great potential to establish itself on the market in the field of modern non-invasive patient monitoring. The main advantage of our solution is the design of a novel patient-friendly measurement probe, which constitutes the core of our system. The functionality of the system was verified by a series of experimental measurements of vital signs (Body Temperature, Heart Rate and Respiratory Rate). The integration of these three vital parameters within one all-purpose probe represents a unique patented solution by the authors. Our experimental results, acquired by testing our novel prototype in research laboratory conditions, have unambiguously proven the functionality of the system. To gain clinical acceptance and find daily utilization in common clinical practice, we are now positioned to carry out extensive clinical research to validate the safety and efficacy of our system.

None of the patients participating in the study complained of any discomfort associated with the presence of the sensor in a thoracic strap placed on her/his chest when asked about it after the examination. The Bland–Altman statistical analysis demonstrates the heart rate detection with a satisfactory accuracy in multiple subjects. For the entire data set, 96.54% of the values lie within the ±1.96 SD range for the HR determination. This fact could be acceptable for clinicians as the sensor is designed for monitoring rather than diagnosis. The results of respiratory measurement are characterized by the maximum relative error of 5.41%, and the maximum relative error of temperature measurement is 0.36%. The clinicians at the University Hospital (Brno, Czech Republic) intend to use this novel sensor in the near future to perform clinical studies on a variety of health care issues.

## Figures and Tables

**Table 1:** Parameters of the fiber Bragg gratings (FBGs) of individual sensors.

Order	Bragg Wavelength (nm)	Left Boundary (nm)	Right Boundary (nm)	Window Spectral Width (nm)
1	1513.444	1512.500	1514.389	1.889
2	1515.735	1514.789	1516.680	1.892
3	1518.028	1517.080	1518.975	1.894
4	1520.324	1519.375	1521.272	1.897
5	1522.622	1521.672	1523.573	1.900
⋮	⋮	⋮	⋮	⋮
32	1585.787	1584.797	1586.776	1.979

**Table 2:** Summary of the experimental results of respiratory measurements.

Patient	M1	M2	M3	M4	M5	M6	F1	F2	F3	F4	Total
Rec. time (mm:ss)	19:10	23:45	18:50	9:30	11:20	12:15	8:30	14:40	17:20	16:30	159:40
Mean respiratory rate (rpm)	17.7	15.0	19.0	15.8	16.2	14.8	15.7	13.9	17.5	16.6	16.22
Number of sample reference	339.3	356.3	357.8	150.1	183.6	181.3	133.5	203.9	303.3	273.9	2482.9
Number of sample sensor	327.2	350.0	339.0	153.9	176.7	172.4	128.4	197.9	286.9	261.2	2386.1
Absolute error	12.01	6.23	18.82	3.80	6.90	8.88	5.07	5.97	16.41	12.65	96.8
Relative error (%)	3.54	1.75	5.26	2.53	3.76	4.90	3.80	2.93	5.41	4.62	3.9

**Table 3:** Summary of the experimental results of heart rate measurements for six men (M1–M6) and four women (F1–F4).

Patient	M1	M2	M3	M4	M5	M6	F1	F2	F3	F4	M1	Total
Rec. time (s)	60	60	60	60	60	60	60	60	60	60	720	1320
Mean heart rate (bpm)	68	72	78	75	79	73	81	84	79	87	88	1832
Error	3	3	2	2	4	2	2	3	4	2	32	59
Samples in												
±1.96 SD (%)	95.58	95.83	97.43	97.33	94.93	97.26	97.53	96.42	94.93	97.70	96.96	96.54

**Table 4:** Summary of the experimental results of body temperature measurements.

	Time (s)	30	60	90	120	150	180	Mean (s)	Relative Error (%)
Male 1	Ref (°C)	35.6	35.6	35.7	35.7	35.7	35.7	35.7	0.28
	Sen (°C)	35.6	35.7	35.9	35.8	35.8	35.8	35.8	
Male 2	Ref (°C)	35.4	35.6	35.7	35.7	35.7	35.7	35.6	0.28
	Sen (°C)	35.2	35.5	35.5	35.6	35.7	35.7	35.5	
Male 3	Ref (°C)	36.3	36.4	36.5	36.5	36.5	36.5	36.5	0.28
	Sen (°C)	36.1	36.3	36.4	36.5	36.5	36.5	36.4	
Male 4	Ref (°C)	36.4	36.4	36.6	36.7	36.7	36.7	36.6	0.18
	Sen (°C)	36.3	36.3	36.5	36.6	36.7	36.7	36.5	
Male 5	Ref (°C)	36.6	36.7	36.7	36.7	36.7	36.7	36.7	0.18
	Sen (°C)	36.4	36.5	36.7	36.7	36.7	36.7	36.6	
Male 6	Ref (°C)	36.2	36.3	36.3	36.3	36.3	36.3	36.3	0.18
	Sen (°C)	36.0	36.2	36.3	36.3	36.3	36.3	36.2	
Female 1	Ref (°C)	36.7	36.8	36.8	36.8	36.8	36.8	36.8	0.14
	Sen (°C)	36.5	36.6	36.7	36.8	36.8	36.8	36.7	
Female 2	Ref (°C)	36.9	36.9	37.0	37.0	37.0	37.0	37.0	0.23
	Sen (°C)	36.6	36.7	36.9	36.9	36.9	37.0	36.8	
Female 3	Ref (°C)	36.2	36.3	36.3	36.3	36.3	36.3	36.3	0.36
	Sen (°C)	36.0	36.1	36.2	36.3	36.3	36.3	36.2	
Female 4	Ref (°C)	35.9	36.0	36.1	36.1	36.1	36.1	36.1	0.23
	Sen (°C)	35.7	35.9	36.0	36.0	36.1	36.1	36.0	
